# Pigmentary retinal dystrophy associated with peroxisome biogenesis disorder-Zellweger syndrome spectrum

**DOI:** 10.1093/omcr/omae067

**Published:** 2024-06-07

**Authors:** Henry Zou, Liliya Sutherland, Brooke Geddie

**Affiliations:** Michigan State University College of Human Medicine, 15 Michigan St NE, Grand Rapids, MI 49503, United States; Retina Specialists of Michigan, 15 Michigan St NE, Grand Rapids, MI 49503, United States; Pediatric Ophthalmology, Helen DeVos Children’s Hospital, 15 Michigan St NE, Grand Rapids, MI 49503, United States

**Keywords:** pigmentary retinal dystrophy, retinitis pigmentosa, peroxisome biogenesis disorder-Zellweger syndrome spectrum

## Abstract

Pigmentary retinal dystrophy (PRD) is a group of inherited disorders involving the progressive degeneration of rod and cone photoreceptors and the retinal pigment epithelium (RPE), which can progress to pigmentary retinopathy (PR). We present a case of PRD in a female pediatric patient who has pathogenic variants in the PRPH2 and PEX1 genes. The patient has associated macular edema and secondary visual impairment. Treatment has included serial dexamethasone intravitreal implant injections and topical dorzolamide. The PEX1 gene mutation is associated with peroxisome biogenesis disorder-Zellweger syndrome spectrum (PBD-ZSS) and resulting retinal dystrophies. The PRPH2 mutation may play a role in macular edema and PRD, as it is implicated in macular degeneration, choroid defects, and photoreceptor dysfunction. In this case, we review multiple gene mutations playing potential etiologic roles for PRD and discuss care management.

## Introduction

Pigmentary retinal dystrophy (PRD) encompasses a group of inherited retinal disorders characterized by progressive degeneration of photoreceptors and retinal pigment epithelium (RPE) [1]. The onset varies from early childhood to middle age. In retinitis pigmentosa (RP), rods are the first photoreceptors to degenerate leading to peripheral vision loss and night blindness in the early stages of the disease [2, 3]. Rod-cone degeneration occurs during the advanced stages of RP, in which cones experience degeneration with resulting central vision loss, color vision impairment, and sensitivity to glare [4]. However, cones are primarily affected in certain cone-rod pigmentary retinal dystrophies or cone dystrophies, whereas rods are either not affected or affected later [4, 5]. Fundoscopic clinical features include intraretinal pigment migration, optic disc pallor, and attenuated retinal vasculature [3]. If PRD progresses to pigmentary retinopathy (PR), RPE mottling and disrupted RPE clumping into bone-spicule formations can be seen in addition to the previous findings [3]. Depending on the genetic etiology of the retinal dystrophy, patients can present with legal blindness at birth, in adolescence, or by 40–50 years of age [1]. However, only 0.46% of retinitis pigmentosa patients progress to total blindness or no light perception [6].

PRD can be inherited in autosomal dominant, autosomal recessive, and X-linked recessive patterns; multiple genetic defects have been identified as triggers in each inheritance pattern [3]. PRD has also been associated with mitochondrial cytopathies [4]. There are over 120 gene mutations that have been identified as resulting in PRD [7]. We present a case of PRD in a female pediatric patient found to have pathogenic variants in the PRPH2 and PEX1 genes.

## Case Report

**Figure 1:**
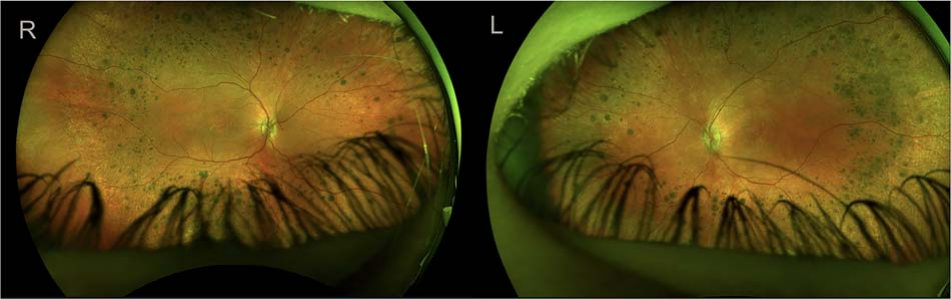
Ultrawide field photo of both eyes showing pallorous disc halo, attenuated arteries, blunted foveal reflex, and coarse pigment clumps in the periphery.

**Figure 2:**
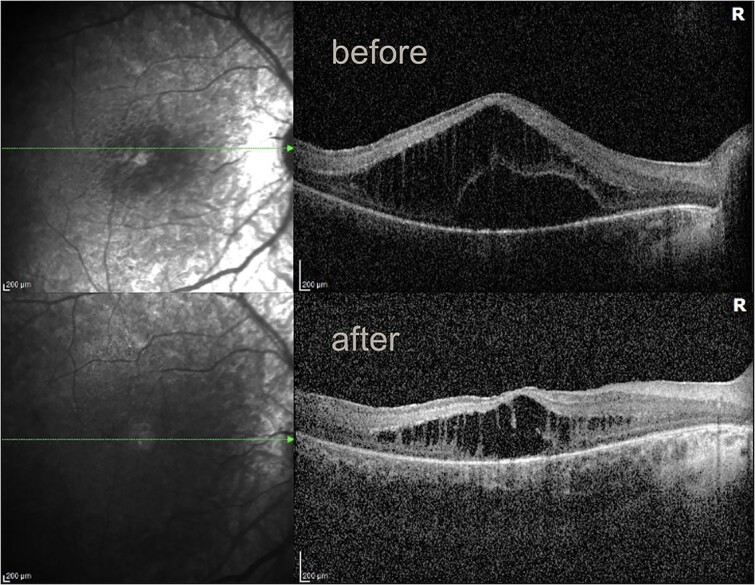
OCT of the right eye before and after intravitreal dexamethasone implant showing reduced macular edema (age 4).

**Figure 3:**
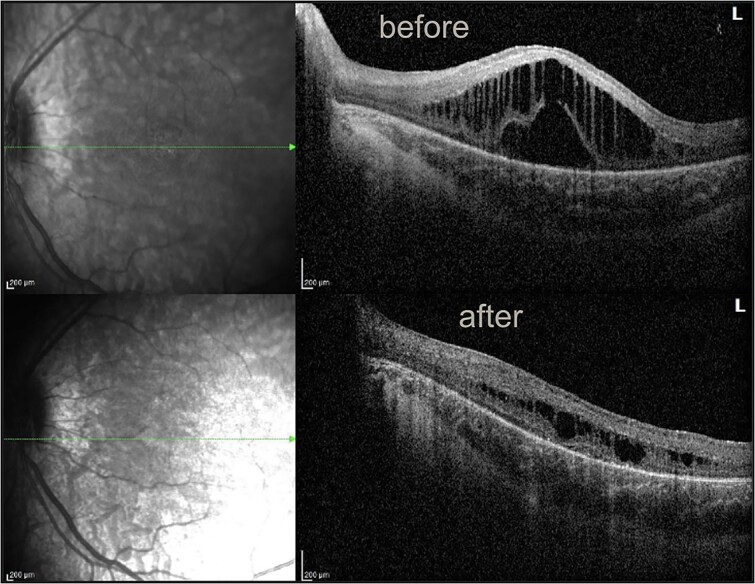
OCT of the left eye before and after intravitreal dexamethasone implant showing reduced macular edema (age 4).

A 4-year-old female patient presented to the pediatric ophthalmologist with concern for developmental delay, esotropia, abnormal eye movements, and ‘feeling around for objects’ despite full-time glasses wear. Further medical history included hearing impairment, developmental delay with hypotonia, small stature for age, and dental enamel changes. A comprehensive evaluation revealed visual acuity of 5/80 in both eyes, esotropia, sensory cyclovertical nystagmus, hyperopia with astigmatism, extensive retinal pigmentation 360° in the mid-periphery (Fig. 1), and decreased foveal reflexes concerning for macular edema bilaterally. Optical Coherence Tomography of the macula revealed thickened retinal segments and schisis/edema of the outer retinal segments (Figs 2 and 3).

Initial treatment for severe macular edema included topical dorzolamide twice daily bilaterally. However progressive macular edema prompted intravitreal dexamethasone 0.7 mg implant (Ozurdex) injection bilaterally. This resulted in some successful improvement in macular edema and vision (Figs 2 and 3). The most recent best corrected visual acuity was 20/125 in the right eye and 20/125 in the left eye. Her most recent cycloplegic refraction was +4.00 + 1.50 × 115 in the right eye, and +5.00 + 1.50 × 90 in the left eye. Serial intravitreal dexamethasone 0.7 mg implant (Ozurdex) injections bilaterally every 6 months have been required.

The patient was also diagnosed with bilateral sensorineural hearing loss. Subsequent genetic evaluation yielded a pathogenic homozygous mutation for the PEX1 c.2528G>A, p. (Gly843Asp) gene and a heterozygous mutation for the PRPH2 c.806C>G, p. (Thr269Arg) gene which was a variant of unknown significance. No non-acute liver function tests or coagulation studies were available for review.

## Discussion

This patient demonstrates peroxisome biogenesis disorder-Zellweger syndrome spectrum (PBD-ZSS), confirmed by pathogenic mutation of the PEX1 gene. PEX1 gene mutations account for 70% of PBD-ZSS cases, which display clinical features including hypotonia, developmental delay, vision and hearing impairment, enamel hypoplasia of secondary dentition, nail abnormalities, and liver dysfunction [8]. PBD-ZSS-associated retinal dystrophy is due to impaired endogenous synthesis of docosahexaenoic acid (DHA), which is vital to brain and retinal development and function [8]. Ocular findings in historical cases of PBD-ZSS have consistently included loss of photoreceptors, RPE, and ganglion cells [8, 9]. Retinal pigment dispersion, optic disc pallor and hypoplasia, and narrowed retinal arterioles were also consistently observed [9]. PBD-ZSS has less consistently been associated with cataracts, glaucoma, and corneal clouding [8, 9].

This patient also demonstrates a heterozygous mutation for PRPH2. This mutation may contribute as well to our patient’s ocular findings. The PRPH2 mutations (Peripherin2) have been implicated in macular degeneration, neural retinal degeneration, RPE atrophy, and choroid defects [10]. Peripherin2 is a photoreceptor-specific glycoprotein that is essential for the proper formation of rod and cone outer segments specialized for phototransduction [10].

We present a case of PRD in a female pediatric patient who has mutations in the PRPH2 and PEX1 genes. This case demonstrates the potential for multiple gene etiologies in a single patient with PRD and the significant resulting visual impairment. However, potential gene-targeted treatment in the future will be beneficial, including adeno-associated virus 9-mediated gene augmentation therapy and cell-type transplantation for PBD-ZSS patients [8].

## Conflict of interest

The authors do not report any conflicts or interest or financial disclosures.

## Funding

None.

## Ethical approval

Waived for case reports.

## Consent

Informed consent was obtained from the patient’s legal guardian/medical proxy for the publication of this case report and its accompanying images.

## Guarantor

Henry Zou.
